# Characterization of Bioactivity of Selective Molecules in Fruit Wines by FTIR and NMR Spectroscopies, Fluorescence and Docking Calculations

**DOI:** 10.3390/molecules28166036

**Published:** 2023-08-12

**Authors:** Young-Mo Kim, Martyna Lubinska-Szczygeł, Yong-Seo Park, Joseph Deutsch, Aviva Ezra, Patraporn Luksrikul, Raja Mohamed Beema Shafreen, Shela Gorinstein

**Affiliations:** 1 Industry Academic Collaboration Foundation, Kwangju Women’s University, Gwangju 62396, Republic of Korea; bliss0816@kwu.ac.kr; 2 Department of Analytical Chemistry, Faculty of Chemistry, Gdansk University of Technology, 80-233 Gdansk, Poland; 3 Department of Horticultural Science, Mokpo National University, Muan 58554, Republic of Korea; ypark@mokpo.ac.kr; 4 Faculty of Medicine, Institute for Drug Research, School of Pharmacy, The Hebrew University of Jerusalem, Jerusalem 9112001, Israel; josephd@ekmd.huji.ac.il (J.D.); aviva.friedman-ezra@mail.huji.ac.il (A.E.); 5 Department of Chemistry, Faculty of Science, Kasetsart University, Bangkok 10900, Thailand; luksirikul.p@gmail.com; 6 Center for Advanced Studies in Nanotechnology for Chemical, Food and Agricultural Industries, KU Institute for Advanced Studies, Kasetsart University, Bangkok 10900, Thailand; 7 Dr Umayal Ramanathan College for Women, Alagappa University, Alagappapuram, Karaikudi 630003, Tamilnadu, India

**Keywords:** kiwifruit, pomegranate, persimmon, wine, antioxidant activity, total phenolics, human serum proteins, binding properties, health benefits, docking calculations

## Abstract

Fourier transform infrared (FTIR) and proton nuclear magnetic resonance (^1^H NMR) spectroscopies were applied to characterize and compare the chemical shifts in the polyphenols’ regions of some fruit wines. The obtained results showed that FTIR spectra (1800–900 cm^−1^) and ^1^H NMR (δ 6.5–9.3 ppm) of different fruit wines can be used as main indices of the year of vintage and quality of fruit wines. In addition to the classical determination of antioxidant profiles and bioactive substances in wines, fluorometric measurements were used to determine the interactions of wine substances with the main human serum proteins. The results showed relatively high binding properties of wines with the highest one for pomegranate, followed by kiwifruit and persimmon wines. The interactions of vitamin C, catechin and gallic acid with human serum albumin (HSA) were also examined by docking studies. The docking calculations showed that gallic acid has a stronger binding affinity compared to catechin and vitamin C. The stronger binding affinity of gallic acid may be due to three hydrogen bonds and pi–pi interactions. The fluorescence and docking studies proved that only the bioactive compounds of wines and not the amount of alcohol have high binding properties to human serum proteins. The emphasis in this report was made on the utility of FTIR, NMR and fluorescence of wines as a mean of wine authentication and its fingerprint. The findings, based on polyphenols from fruits and fruit wines, their bioactivity and health properties, offer valuable insights for future endeavours focused on designing healthy food products.

## 1. Introduction

Most of the traditional, citrus and tropical fruits (jackfruit, cashew apple, mangoes, papaya, pineapple, litchi, guava, banana, pomegranate, kiwifruit, persimmon and many others) are important sources of antioxidants, vitamins and minerals and form a very healthy part of a diet [1]. The investigations in vitro and in humans showed that red grapefruit positively influences serum triglyceride levels in patients suffering from coronary atherosclerosis [2]. It was shown that the effective utilization of ripe and overripe fruits, by processing them into fermented beverages, was revealed as a new and promising alternative to grapes. Concerning this, the manufacture of wine from fruits other than grapes was developed in recent years. The use of two different varieties of pawpaw (rose, red and yellow) to produce table wine was reported [3]. Different beverages mostly prevent cardiovascular diseases [4,5,6,7].

In this respect, two fruits growing in South Korea persimmon ‘Fuyu’ and kiwifruit ‘Hayward’ were investigated and compared [8]. The yield of wine production of ‘Hayward’ kiwifruit (*Actinidia deliciosa*) as material for wine production was increased from 63.35% to 66.19% by using ripened kiwifruits. The quality characteristics of kiwifruit wine made from over-ripened fruit treated with pectinase showed higher values of wine in many aspects such as sensory value, alcohol and total phenolics content, antioxidant activity, minerals and production yield. The most important factor is to preserve the antioxidant protective properties in wine products. Phenolic substances are the main antioxidant agents [9]. The application of fruits is wide, including various properties, such as antioxidant, anti-inflammatory, and antiwrinkle in Korean persimmon [10]. Different advanced methods were used for the determination of the quality of fruits and wines. A total of 30 metabolites in (*Passiflora edulis* Sims) juice during maturation and ripening were successfully identified using proton nuclear magnetic resonance (^1^H NMR), the majority of which belonged to primary metabolites, consisting of 14 amino acids, seven sugars and six organic acids, but mostly dealing with aliphatic part of the shifts [11]. A ^1^H NMR-based metabolomic approach was used for conventionally and organically grown pomegranate fruits, using most essential amino acids, organic acids and phenolic content [12]. Such measurements were used for grape berries [13,14]. A combination of Fourier-transform–near-infrared spectroscopy (FT-NIR) and ^1^H nuclear magnetic resonance (NMR) spectroscopy was used to discriminate wines containing anthocyanins that originated from black rice and grape wine with the main purpose to detect adulteration of wine [15].

Recently, different analytical methods were reported for the characterization of fruit wines, based on selected parameters [16]. A new sensor can be commercialized and deployed for monitoring gallic acid in wine matrices and fruit juices [17]. Despite a high number of reports on the quality of fruit wines, there is a lack of knowledge on how the wine bioactive compounds react with human serum proteins and the use of advanced analytical methods for the determination of their quality. We hypothesized that if wines show high binding properties with the main human proteins, then these substances can be careers for drugs as well in human metabolism. As can be seen, the most cited research reports were only on white and red wines from grapes, but even one report on fruit wines does not exist. Our aim was to introduce similar analytical methods for fruit wines, which are widely used for traditional ones, and to compare the obtained results with the data shown in the literature. To our knowledge, we are the first to deal with this matter.

In order to cover the above, this study compared some fruit wines using the most advanced analytical methods such as FTIR, ^1^H NMR and fluorescence measurements to characterize and compare the quality of wines.

## 2. Results and Discussion

### 2.1. Bioactivity of Wine Samples

The obtained results of bioactive compounds in fruit wines showed that persimmon had lower results than pomegranate and kiwi (Table 1). The amount of polyphenols in pomegranate was in line with recent reports, where phenolics were in the range of 3578–5108 mg/100 g [12].

A significant variation in total phenolic content was found among the crops of pomegranate cultivars. The differences in phenolic compounds can be due to genetic diversity and environmental conditions [18]. The estimation of kiwi wine was twice as high, as shown in recent reports, where the amount of polyphenols varied in the range of 772 mg/L [1]. Hence, this result might be attributed to the release of phenols contained in pomace, which were positively correlated with the antioxidant activities of kiwi wine. The concentrations of epicatechin, catechin and caffeic acid were relatively higher, followed by gallic and other phenolic acids [19]. Some reports showed that the use for winemaking ripened and over-ripened fruits with the use of enzymes showed higher quality of wines. The main indices of wines such as volatile and phenolic compounds reflected the quality of obtained wines [7,9,20]. There was a similar correlation between total polyphenols, tannins, vitamin C and values of antioxidant activities in fruit wines prepared from kiwi and persimmon and in the fresh kiwi ‘Hayward’ and persimmon ‘Fuyu’ [8,10,14,20,21]. The value of polyphenols in pomegranate wine was 1707.3 mg/L (Table 1), showing the same estimation as declared values of 2270 mg/100 g and 1651 mg/100 g for organic and conventional pomegranate juices, respectively [12].

Total antioxidant capacities (mmol TE/L) of pomegranate, kiwi and persimmon wines, determined by ABTS, were 20.2, 16.5 and 11.4 and by FRAP—7.4, 5.9 and 3.4, respectively, and corresponded with their bioactive contents. The values of persimmon wines by ABTS were 2.0–4.1 mmol/L, showing lower values in the reported literature by other researchers, but they showed the same order of scavenging activity, which depends on the temperature of fermentation. These results indicated that high fermentation temperature had the potential to produce wine with the preferable antioxidant ability [21,22]. The temperature produced great variations in fermentation and final wine quality. High fermentation temperature accelerated sugar consumption and alcohol formation and enriched the persimmon wine with phenolics, tannins and flavonoids [7]. The anthocyanins in the pomegranate varied greatly with the cultivars, maturity level, climate conditions of production area and seasonal variations in weather conditions (5.91–80.70 mg/100 g). Cyanidin 3,5-diglucoside and delphinidin 3,5-diglucoside are the major anthocyanins in pomegranate juice [23]. The antioxidative activity measured by 2,2-diphenyl-picrylhydrazyl (DPPH) was correlated with total phenolic contents but not with juice anthocyanins [18]. High levels of phenolic compounds, anthocyanins and ascorbic acid 7.15–10.61 (mg/100 mL) make pomegranate juice an excellent natural resource of antioxidants.

Studies revealed antimicrobial, antiviral, anticarcinogenic and anti-inflammatory activities of the juice [20,23,24,25]. The ascorbic acid was significantly different among Italian and Iranian pomegranate genotypes and ranged from 89.0 to 236.3 mg/L and showed the highest levels than the wine (Table 1) [26,27].

### 2.2. FTIR Spectroscopy

**Figure 1:**
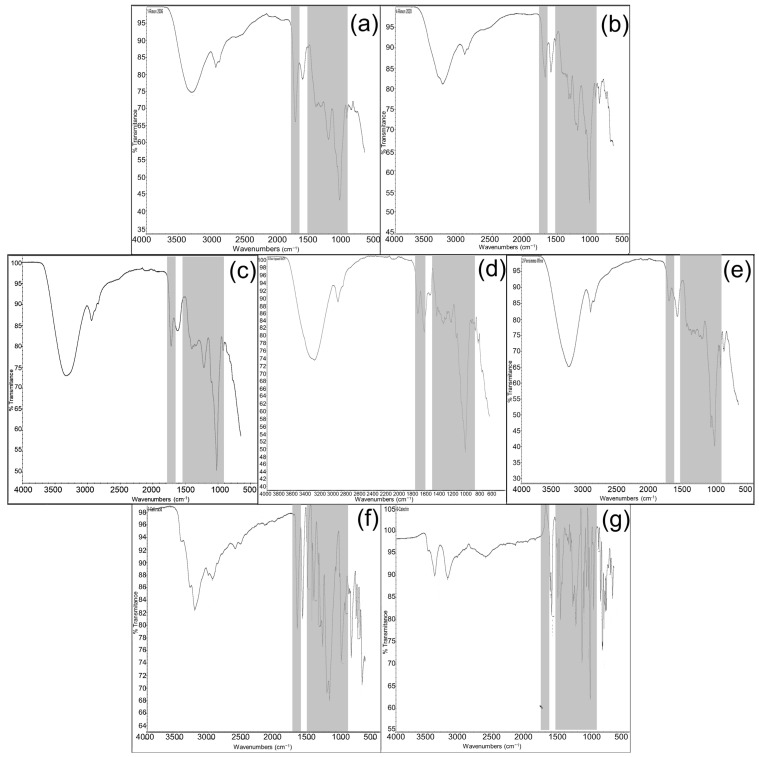
FTIR spectra of (**a**–**g**)**,** pomegranate of vintage 2006 (Pomeg2006); pomegranate of vintage 2020 (Pomeg2020); persimmon 2022; kiwifruit 2022; pomegranate of vintage 2022 (Pomeg2022) wine extracts; gallic acid and catechin measured in the range of 4000–500 cm^−1^ with a resolution of 2 cm^−1^ and 32 scans.

**Figure 2:**
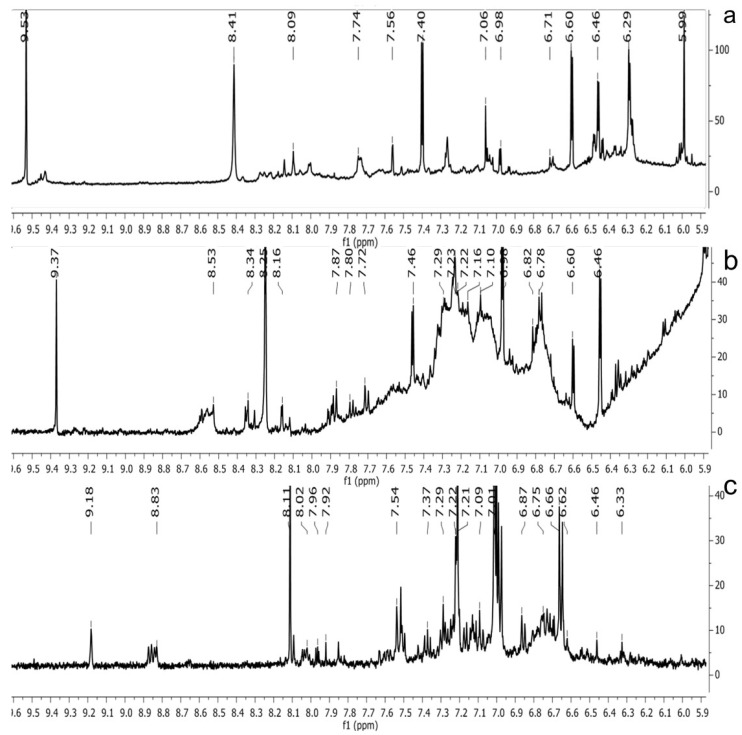
^1^H NMR spectra of the methanol extract from (**a**)**,** pomegranate; (**b**), persimmon; (**c**)**,** kiwifruit wines. The spectra obtained at 500 MHz were scaled in accordance with TSP used as the internal standard.

The obtained data of wine FTIR spectra are shown in Figure 1. As was mentioned above, two additional samples of pomegranate fruit wines of different vintages 2006 (Pomeg2006) and 2020 (Pomeg2020) were used in order to show the differences and identity in FTIR spectra of pomegranate wine from vintage 2022 (Pomeg2022). Two main regions were used in the estimation of obtained spectroscopic results. The first chosen region was from 3400 to 2900 cm^−1^. All investigated samples showed two peaks in the first region from 3400 to 2900 cm^−1^: Pomeg2006 (Figure 1a, 3312 cm^−1^, 2937 cm^−1^); Pomeg2020 (Figure 1b, 3276 cm^−1^, 2913 cm^−1^); Pomeg2022 (Figure 1e, 3333 cm^−1^, 2930 cm^−1^); kiwifruit (Figure 1d, 3286 cm^−1^, 2930 cm^−1^) and persimmon (Figure 1e, 3276 cm^−1^, 2936 cm^−1^). The used standard gallic acid (Figure 1f, 3267 cm^−1^, 2997 cm^−1^) showed similar peaks as all wine samples, but catechin (Figure 1g, 3405–3204 cm^−1^, 2619 cm^−1^) was different. The smaller peak at 2937 cm^−1^ was more specific for all wine samples. The estimated data were in line with the reported results [28,29], explaining that the band at 3300 cm^−1^ was designated as the O–H stretching vibration in the polyphenols. The small signals around 2960 and 2910 cm^−1^ in all samples were originated from the C–H stretch vibration in the aromatic methoxy and in the methylene groups of side chains. The most relevant second region was between 1770 and 1650 cm^−1^ and was used as a fingerprint region. So, the following spectra, deriving from wine samples, were estimated: for Pomeg2006 (Figure 1a, 1725 cm^−1^, 1716 cm^−1^, 1700 cm^−1^, 1602 cm^−1^); Pomeg2020 (Figure 1b, 1699 cm^−1^); Pomeg2022 (Figure 1e, 1725 cm^−1^, 1721 cm^−1^, 1700 cm^−1^, 1622 cm^−1^); kiwi (Figure 1d, 1727 cm^−1^, 1725 cm^−1^ 1700 cm^−1^, 1632 cm^−1^), persimmon (Figure 1c, 1725 cm^−1^ 1723 cm^−1^, 1700 cm^−1^, 1595 cm^−1^). The used standards were gallic acid (Figure 2f, 1700 cm^−1^, 1695 cm^−1^) and catechin (Figure 1g, 1631 cm^−1^). The similarity in the number of peaks and their position was shown nearly in all samples and standards. The obtained results were in the same line as in other reports [30]. The peak at 1725 cm^−1^ was assigned to the carbonyl C=O stretching band of protonated carboxylic acid, characteristic of the galloyl unit of hydrolyzable tannins [31]. The large peak occurred between 1700 and 1560 cm^−1^ corresponding to C–O band and potentially overlapping with amide bands at 1650 cm^−1^. A shoulder around 1700 cm^−1^ was due to the stretching of the carbonyl C=O group. The band around 1630-1442 cm−^1^ (Figure 1d,f,g) had an evident signal, which belonged to the skeleton vibration of the benzene nucleus. A peak at 1602 cm^−1^ and those at 1442 cm^−1^ were due to the C––C–C stretching, typical of aromatic systems. Peaks around 1618 cm^−1^ were assigned to the -COO- stretching.

The next important region was between 1540 and 900 cm^−1^. The following spectra, deriving from wine samples, were estimated for: Pomeg2006 (Figure 1a, 1205 cm^−1^, 1035 cm^−1^, 1016 cm^−1^); Pomeg2020 (Figure 1b, 1540 cm^−1^, 1335 cm^−1^, 1200 cm^−1^, 1020 cm^−1^, 1016 cm^−1^); Pomeg2022 (Figure 1e, 1213 cm^−1^, 1036 cm^−1^, 1016 cm^−1^); kiwi (Figure 1d, 1442 cm^−1^, 1223 cm^−1^, 1016 cm^−1^, 1014 cm^−1^), persimmon (Figure 1c, 1211 cm^−1^, 1020 cm^−1^, 1016 cm^−1^). The used standard gallic acid (Figure 2f, 1539 cm^−1^, 1239 cm^−1^, 1018 cm^−1^, 1016 cm^−1^) and catechin (Figure 1g, 1631 cm^−1^, 1512 cm^−1^, 1235 cm^−1^, 1040 cm^−1^, 1016 cm^−1^). From 1540 to 900 cm^−1^ (C–C absorption bands, C–O vibrations, C–OH bending deformation, C–H bond stretching and C=O and C=C groups) appeared [32]. The major protein bands included amide II (C–N stretching coupled with N–H bending) vibrations at approximately 1540 cm^−1^. Peaks at 1540 cm^−1^ and 1442 cm^−1^ belonged to the C–-C–C of aromatic rings. The spectra showed –CH bending and –CH_2_ wagging at 1335 cm^−1^. Peaks at 1046 cm^−1^ and 1024 cm^−1^ were ascribed to the C–OH stretching in glycosylated phenols. Peaks at 1020 cm^−1^ were assigned to anthocyanins. The peak around 1016 cm^−1^ was ascribed to the phenolic C–OH in all investigated samples including standards. For signals with wavelengths smaller than 900 cm^−1^, the aromatic CH stretching vibration was detected (Figure 1b, 866 cm^−1^); Figure 1d (862 cm^−1^, 820 cm^−1^); Figure 1e (873 cm^−1^), Figure 1f (865–697 cm^−1^) and Figure 1g (889–673 cm^−1^). Similar bands were estimated for comparison of persimmon and kiwifruit [8]. The band around 877 cm^−1^ (Figure 1b,d–f) was related to C—C stretching vibration of organic molecules.

It was interesting to compare the spectra of investigated samples (Table 2).

The comparison of different ranges of FTIR spectra between pomegranate and kiwifruit wines in the ranges of 3628–3286 cm^−1^, 1205–1113 cm^−1^ and 981–906 cm^−1^ were relatively low, but at 1095–1054 cm^−1^, where the main polyphenol peak appeared, the correlation was about 0.81. The estimation of pomegranate and persimmon wines in the ranges of 3086–2906 cm^−1^, and 1740–1569 cm^−1^ showed a low comparison of the peaks. Oppositely, the ranges of 1314–1222 cm^−1^, 1126–1038 cm^−1^ and 1012–907 cm^−1^ showed high similarity of peaks from 0.81 to 0.88. The comparison of kiwifruit and persimmon wines showed low similarity in the ranges of 3569–3138 cm^−1^, 1823–1665 cm^−1^ and 1284 and 1139 cm^−1^ (Table 2). This comparison shows the correlation between the polyphenol region, which shows the antioxidant properties of the wines [32,33].

As was shown, the main region of the FTIR spectra was in the range of 3400–2900 cm^−1^. Then, the most important was between 1770 and 900 cm^−1^, dividing into sub-regions as 1770–1650 cm^−1^ and 1540–900 cm^−1^ and a minor one of 900–700 cm^−1^. These results were in line with other reports where it was shown that in the spectral region 900–1800 cm^−1^, a good determination capability for total phenolic and flavonoid composition was observed [28]. The same spectral region of wines was measured by ATR-FTIR and UV–Vis to investigate the fingerprint region of polyphenols. The method was able to identify peaks correlated with anthocyanins and flavanols for the red wines, non-flavonoids and flavonoids for the white wines, and glycosylated phenolics for both wines. The obtained results were in accordance with recently published reports [34,35]. The determination capability of FTIR spectroscopy was investigated by analyzing the same spectral region as in the present report, 950–1821 cm^−1^, deriving for wine samples aged in different wooden barrels [36]. The use of FTIR spectra from samples of ‘Cabernet Sauvignon’ Mexican wines showed a proposed model for the determination of total bioactive phenolic compounds and antioxidant capacities, and the spectral area used was 824–1550 cm^−1^ [37].

In many cited reports using FTIR spectroscopy, the interest was focused on the 1600–900 cm^−1^ spectral region, because in this area, characteristic groups absorb and the ‘fingerprint’ region is important; in this region, any differences between the spectra can be detected, as was shown in the investigated samples. Generally, in the 1600–900 cm^−1^ region, bands originating from wine phenols can be found [36,38,39]. It is known from the literature that most of the bands originating from wine polyphenols can be found in the spectral region 1750–950 cm^−1^. The fingerprint region was nearly the same in many reports; some used two areas: 1800–1500 cm^−1^ and 1300–900 cm^−1^ of their FTIR spectra [40]. Other reports estimated the region from 1800 to 900 cm^−1^ as the fingerprint region and showed differences between the wine samples. These were caused by the vibration of the C–O, C–C, C–H and C–N bonds [41,42]. The obtained results of FTIR spectra of investigated samples of fruit wines were in a similar fingerprint as conventional grape wines, showing the presence of phenols. The current reports focused on the use of the most popular spectroscopic techniques applied for wine characterization, authentication and quality control and, as a result, to show their fingerprints.

### 2.3. NMR Spectra

The ^1^H NMR chemical shifts of the assigned compounds in pomegranate, persimmon and kiwi wines are shown in Figure 2 and some are listed in Table 3. The aromatic region (δ 6.5–9.3) of the ^1^H NMR spectra of wine extracts exhibited characteristic signals arising from the phenolic content of the wines such as gallic acid—δ 7.06, 7.10 and 7.09 for pomegranate, persimmon and kiwi wines (Figure 2). Some peaks were similar in all wine samples (6.60; 6.62–7.56; 7.72–8.16). Persimmon wine (Figure 2b, Table 3) showed relatively high peaks at 8.35 and 9.37 ppm, similar to the kiwifruit wine (Figure 2c, Table 3), and the peaks were at 8.11 ppm and small ones at 8.83 and 9.18 ppm. Most peaks were found for pomegranate and kiwifruit samples at 6.62–7.54 ppm. The flavonoids appeared as quercetin signals at δ 6.29, δ 7.56 and δ 7.54. The results were underlined for the following peaks found for persimmon (δ 7.40- protocatechuic acid; δ 7.06-gallic acid; δ 6.74-catechin [43]). In particular, almost the entire portion of the signal between 6.60 and 7.01 ppm had a significant role in the classification, as well as the regions, centred around 7.06–7.09, 7.16–7.22 and 7.40–7.46 ppm and minor peaks at 8.41, 8.53 and 8.83 ppm and matched with NMR assignment of anthocyanin-derivatives [12,15]. Anthocyanins, which are responsible for the red colour of wines, were analyzed by 1D NMR spectroscopy in Slovenian wine samples that were similar to pomegranate, according to the colour of wines [44]. The singlet at δ 8.25 ppm (persimmon) was assigned to the phenolics because the proton signals in this range can usually be observed from the aromatic ring [31,45].

The correlation peaks at 7.06 and 7.40 ppm for pomegranate, 7.10 and 7.72 ppm for persimmon and 7.21 and 7.37 ppm for kiwifruit wines were reasonably due to ortho protons in aromatic systems of anthocyanin derivatives or adducts [37,41,42]. These data are in line with the cited literature, where NMR spectroscopy was used for quality control and authentication of grape wines [14,29,44,45,46,47,48]. The most particular part of each ^1^H NMR spectrum was in the δ 6.5–10.5 range, which corresponded to the aromatic rings of the extractable from almost all parts of the pomegranate. Among polyphenols, anthocyanins are particularly abundant in fruits [49,50]. Proton resonances of phenolic compounds including gallic acid, ellagic acid, protocatechuic acid and catechin were identified in the aromatic region (Figure 2). Table 3, which was based on the results of recently cited reports, shows the following chemical shifts: gallic acid was observed at δ 7.04 (s), while a sharp singlet signal at δ 7.47 was assigned to ellagic acid. Catechin and the tannins, α-and β-punicalagins were characterized using 2D NMR spectra. Fumaric acid 6.18 (s); α-punicalagin 7.21 (s), 7.01 (s), 6.88 (s); β-punicalagin 7.24 (s), 7.05 (s), 6.92 (s); pelargonidin−3,5-di-O-glucoside 8.93 (s), 8.14 (d), 6.99 (s), 6.96 (s); delphinidin-3-O-glucoside 8.95 (s), 7.91 (s), 6.88 (d, J = 1.5), 6.71(d, J = 1.5); delphinidin-3,5-di-O-glucoside 8.57 (s), 7.09 (s), 6.81(s), 6.62 (s); cyanidin-3,5-di-O-glucoside 9.25 (s), 8.85 (d, J = 7.8), 8.10 (d, J = 2.0), 6.91(s); quercetin 6.22 (s), 6.40 (s), 7.41 (d, J = 8.4); catechin 2.68 (m), 4.02 (m), 5.63 (d, J = 2.3), 5.66 (d, J = 2.3), 6.71 (J = 8.1); protocatechuic acid 6.94 (d, J = 7.0), 7.23 (dd, J = 8.1, 2.0) [50,51]. Some of these shifts were identified in the investigated samples of the present report.

**Table 3:** ^1^H NMR chemical shifts of the assigned compounds from persimmon, kiwifruit and pomegranate wine and fruit extracts.

No.	Tentative Compound	Structure	Persimmon			Kiwi			Pomegranate		
			+/−	δH(ppm), Multiplicity, J Value (Hz)	Lit.	+/−	δH(ppm), Multiplicity, J Value (Hz)	Lit.	+/−	δH(ppm), Multiplicity, J Value (Hz)	Lit.
1	Phenylalanine	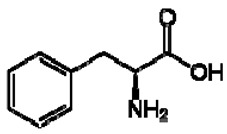	+	7.32, d, 7.4	[52,53,54]	+	7.40, m (2H) 7.35, m 7.30, d, 7.4 (2H)	[45,55]	+	nd	[12]
2	Kaempferol	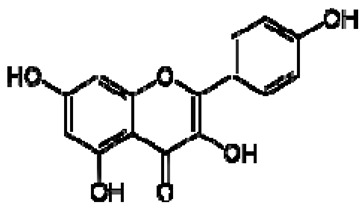	+	8.01, d, 8.0 6.95, d, 8.0 6.32, br d (small d) 6.10, br d (small d)	[55]	+	8.01, d, 8.0 6.95, d, 8.0 6.32, br d (small d) 6.10, br d (small d)	[55]	−	-	-
3	Rutin	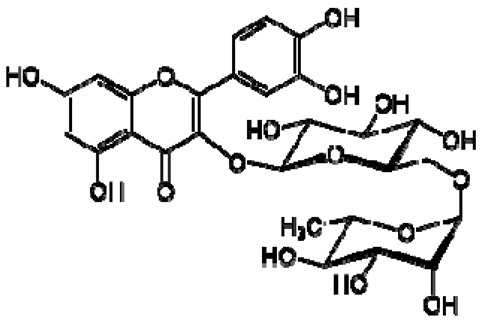	−	-	-	+	7.65, d, 2.0 7.60, dd, 6.82, d, 8.5 6.38, d, 6.19, d, 1.05, d, 7.0 4.51, br s (small d) 5.05, d, 8.0	[45,55]	−	-	-
4	Tryptophan	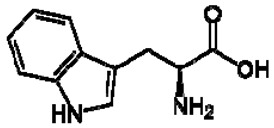	+	7.31	[52,53,54]	+	7.70, d, 8.0 7.54, d, 8.0 7.20, t, 7.0	[45,55]	+	nd	[12]
5	Tyrosine	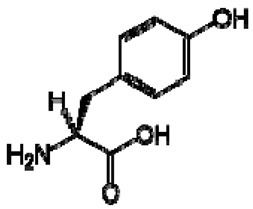	−	-	-	+	3.94, m 7.15, d, 8.0 6.82, d, 8.0	[45,55]	+	nd	[12]
6	Caffeic acid derivatives	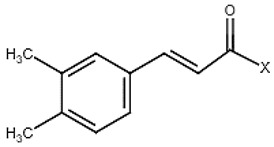	−	-	-	+	7.57, d, 13.0 7.28, br s (small d) 7.22, d, 8.0 6.95, d, 8.0 6.55, d, 13.0	[45,55]	−	-	-
7	Protocatechuic acid	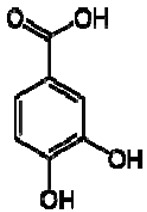	+	7.39, br s (small d) 7.35, br d (dd), 8.0 6.92, d, 8.0	[43,55]	+	7.39, br s (small d) 7.35, br d (dd), 8.0 6.92, d, 8.0	[45]	+	6.94 (d, J = 7.0), 7.23 (dd, J = 8.1, 2.0)	[51]
8	Catechol	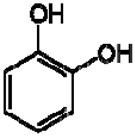	−	-	-	+	6.776.84, m 4.52, d, 7.20 2.94, dd, 15.7, 6.2 2.47, dd, 15.0, 8.0	[45,55]	−	-	-
9	Syringic acid	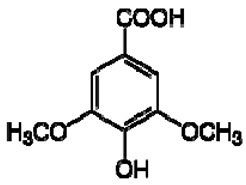	−	-	-	+	7.26, s, 2H 3.89, s	[45]	−	-	-
10	Afzelechin	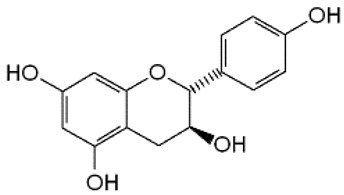	−	-	-	+	2.83, 2.80; 2.79, dd,15.6, 4.8 2.68, d 6.85, d, 8.0 (2H) 7.17, d, 8.0 (2H)	[55]	−	-	-
11	Kaempferol derivatives	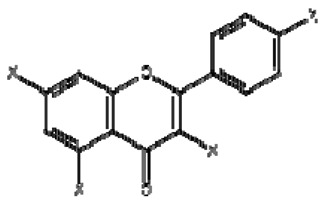	+	6.97, d, 2.7 6.46, d, 2.7	[55]	−	-	-	−	-	-
12	Quercetin derivatives	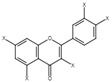	+	7.52, d, 3.5 6.66, d, 3.5	[55]	+	7.52, d, 3.5 6.66, d, 3.5	[45]	−	-	-
13	Gallic acid	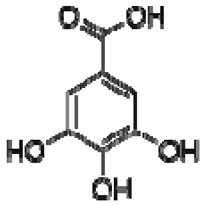	+	7.01 (s)	[43,54,55]	−	-	-	+	7.04 (s)	[20,51]
14	Ellagic acid	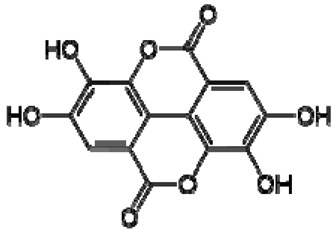	−	-	-	−	-	-	+	7.47 (s)	[51]
15	Punicalagin	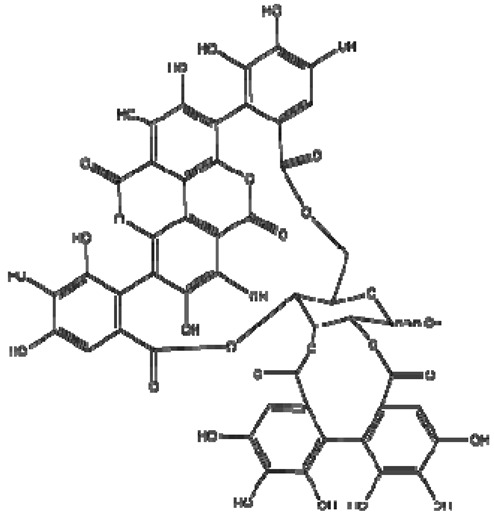	−	-	-	−	-	-	+	(α): 7.21 (s), 7.01 (s), 6.88 (s) (β): 7.24 (s), 7.05 (s), 6.92 (s) 6.53, d 9.89 Hz	[51,56]
16	Pelargonidin-3,5-di-O-glucoside	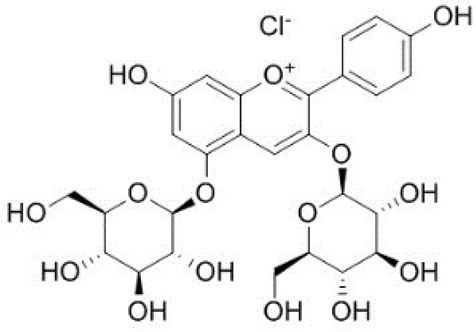	−	-	-	−	-	-	+	8.93 (s), 8.14 (d), 6.99 (s), 6.96 (s)	[51]
17	Delphinidin-3-O-glucoside	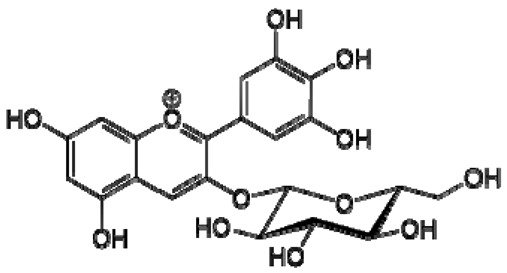	−	-	-	−	-	-	+	8.95 (s), 7.91 (s), 6.88 (d, J = 1.5), 6.71(d, J = 1.5)	[51]
18	Delphinidin-3,5-di-O-glucoside	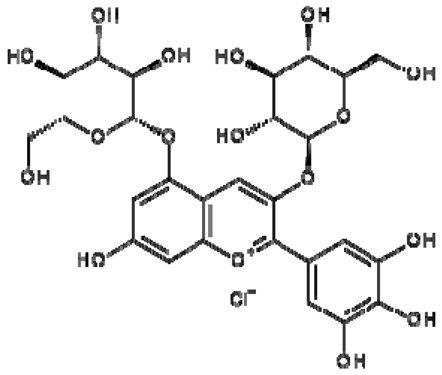	−	-	-	−	-	-	+	8.57 (s), 7.09 (s), 6.81(s), 6.62 (s)	[51]
19	Cyanidin-3,5-di-O-glucoside	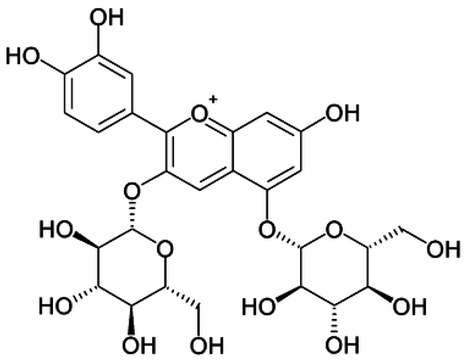	−	-	-	−	-	-	+	9.25 (s), 8.85 (d, J = 7.8), 8.10 (d, J = 2.0), 6.91(s)	[20,51]
20	Quercetin	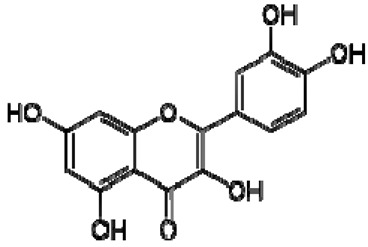	−	-	-	−	-	-	+	6.22 (s), 6.40 (s), 7.41 (d, J = 8.4)	[51]
21	Cyanidin-3-O-glucoside	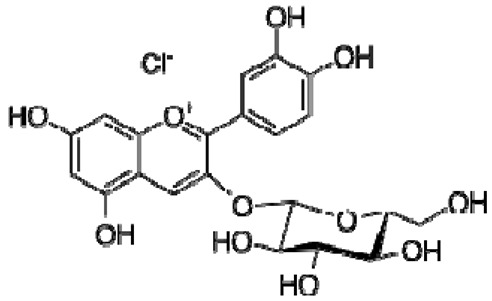	−	-	-	−	-	-	+	nd	[20]
22	Pelargonidin-3-O-glucoside	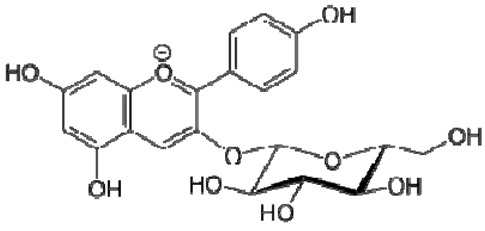	−	-	-	−	-	-	+	nd	[20]
23	*p*-coumaric acid	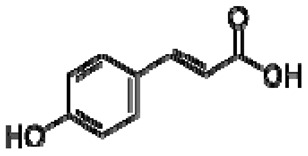	−	-	-	−	-	-	+	nd	[20]
24	Chlorogenic acid	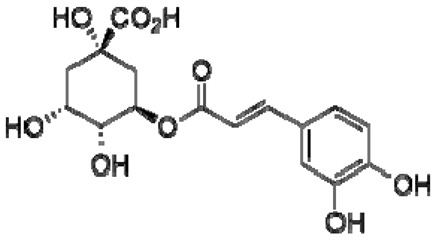	−	-	-	−	-	-	+	nd	[20]
25	Caffeic acid	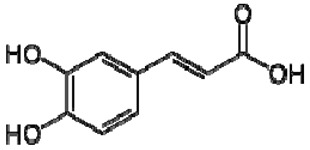	+	7.33 (d, *J* = 16.0 Hz), 7.13 (d, *J* = 1.9 Hz), 7.00 (dd, *J* = 8.0, 2.0 Hz), 6.86 (d, *J* = 8.0 Hz), 6.35 (d, *J* = 16.0 Hz)	[43]	−	-	-	+	nd	[20]
26	Niacin	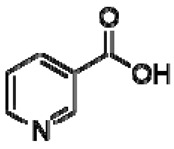	−	-	-	+	8.96, s 8.54, br s 8.25, br s	[45]	−	-	-
27	Hesperidin	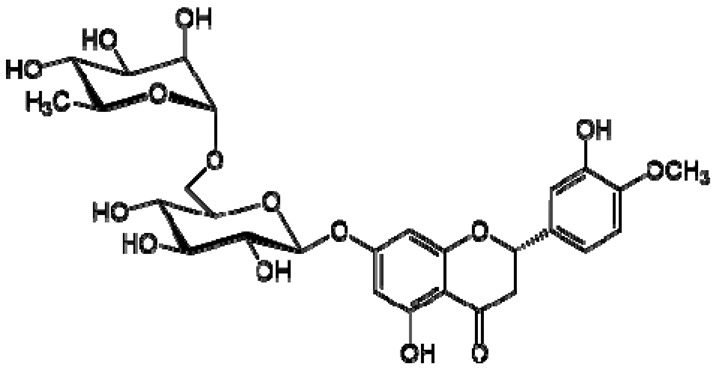	−	-	-	+	6.97, d, 2.7 6.46, d, 2.	[45]	−	-	-
28	Trigonelline	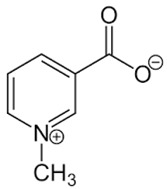	+	9.14 (s), 8.83 (m), 8.07 (m), 4.44 (br s)	[43,52,54]	−	-	-	+	9.12, s; 8.07, t 6.90 Hz	[12,56]
29	Uridine	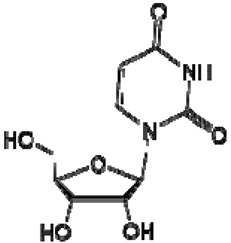	+	7.93 (d, *J* = 7.9 Hz), 5.80 (m), 4.34 (m), 4.22 (m), 4.12 (m), 3.90 (m), 3.80 (m)	[43,52,54]	−	-	-	−	-	-

Abbreviations: s—singlet, d—doublet, t—triplet, dd—doublet of doublets, m—multiplet, br d—broad doublet, br s—broad singlet, nd—not determined.

In exploring the capability of nuclear magnetic resonance spectroscopy for pomegranate juice analysis, the eight aromatic singlet resonances of α- and β-punicalagin were clearly identified in the ^1^H NMR spectra of juice samples (6.7–7.1) [57]. Among polyphenols, anthocyanins are particularly abundant in fruits. Different wine aging conditions were monitored by methods for measurement of antioxidant activity and comparison of 1D and 2D NMR spectra of phenolic species in wines [57,58,59]. As mentioned above, the aim of this research was to find characteristic and main region in FTIR and NMR spectra without quantitative determination of the main compounds. In this report, NMR spectroscopy was applied for qualitative analysis and compositional profiling of wines, especially phenolic compounds. The obtained data of the NMR spectra were in line with recent reports where the identified phenolic compounds were gallic acid, syringic acid, *p*-coumaric acid, *trans*caffeic acid, catechin, epicatechin, ferulic acid, quercetin, kaempferol and trans-resveratrol [32,60]. The fingerprints of NMR spectra were obtained with the emphasis of the aromatic region, which is important for verifying the bioactive substances.

### 2.4. Fluorescence Measurements

**Figure 3:**
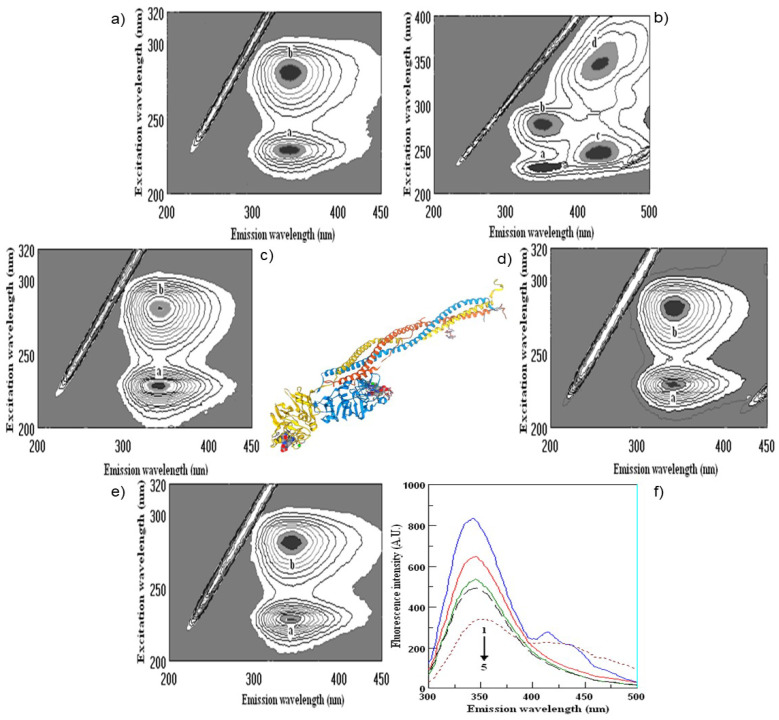
Cross-images of the results obtained from fluorometric measurements in a three-dimensional fluorescence analysis (3D–FL) of the investigated samples after interaction with fibrinogen are shown in the following order: (**a**) fibrinogen (Fgn); (**b**) Fgn + persimmon wine; (**c**) Fgn + pomegranate wine; (**d**) Fgn + kiwifruit wine; (**e**) Fgn + gallic acid; (**f**) (2D–FL) fluorescence intensities (FI) from the top: 1, Fgn in buffer; 2, Fgn + kiwifruit wine; 3, Fgn + persimmon wine; 4, Fgn + gallic acid; 5, Fgn + pomegranate wine with λ_em_ (nm) of 343, 345, 345, 345, 353; fluorescence intensities (FI) of 857.2 ± 13.9; 666.7 ± 11.5; 552.8 ± 8.7; 508.4 ± 6.8; 350.9 ± 5.3 arbitral units (A.U.); **a**, **b**, **c**, **d**, peaks after interaction of investigated samples with human serum proteins.

**Figure 4:**
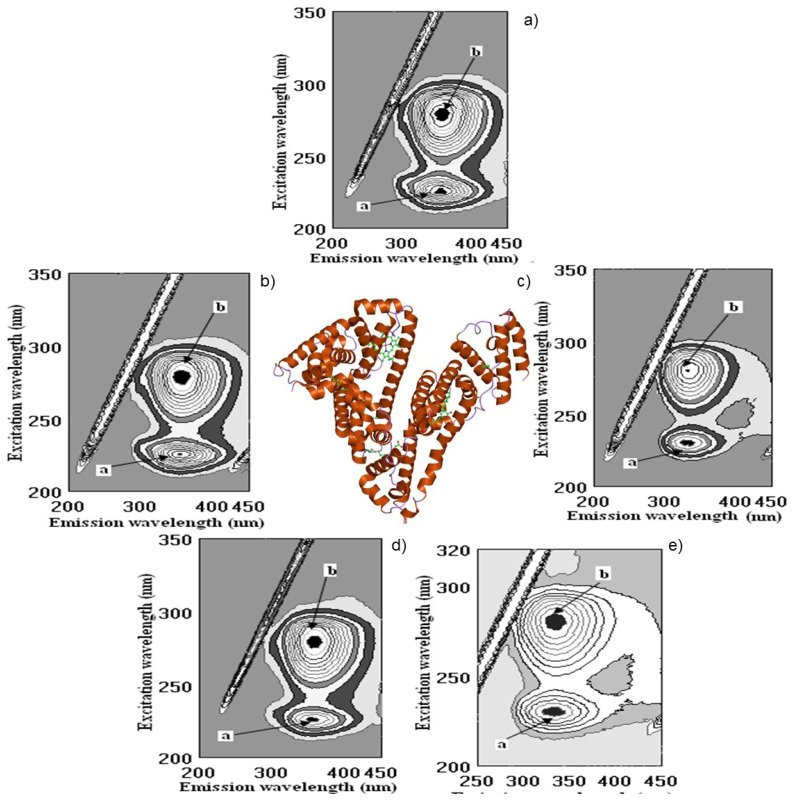
Cross-images of the results obtained from fluorometric measurements in a three-dimensional fluorescence analysis (3D-FL) of the investigated samples after interaction with albumin (Alb) are shown in the following order: (**a**) Alb in the buffer; (**b**) Alb + pomegranate wine; (**c**) Alb + persimmon wine; (**d**) Alb + gallic acid; (**e**) Alb + kiwi wine; **a**, **b**, peaks after interactionof investigated samples with Alb.

**Figure 5:**
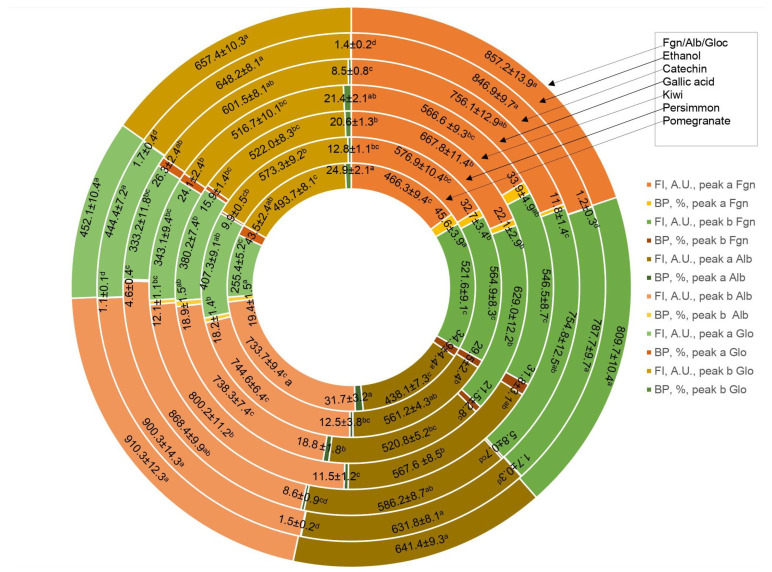
Fluorometric measurements in three-dimensional fluorescence analysis (3D-FL) of persimmon, kiwifruit and pomegranate wines extracts after interaction with human serum proteins fibrinogen (Fgn), albumin (Alb) and globulin (Glo). Abbreviations: FI, fluorescence intensity, A.U., arbitral units. The values of fluorescence intensity for used human serum proteins before interaction with extracted wine samples were the following: FI Fgn of peak **a** (A.U.) = 857.2 ± 13.9; FI Fgn of peak **b** (A.U.) = 809.7 ± 10.4; FI Alb of peak **a** (A.U.) = 641.4 ± 9.3; FI Alb of peak **b** (A.U.) = 910.3 ± 12.3; FI Glo of peak **a** (A.U.) = 452.1 ± 10.4; FI Glo of peak **b** (A.U.) = 657.4 ± 10.3. The locations of peaks **a** and **b** are shown in Figure 3, Figure 4, Figure 5 and Figure 6 (for interpretation of the references to color in this figure legend, the reader is referred to the web version of this article). Values are means ± SD of 5 measurements; means with the different superscripted letters a–d are statistically different.

**Figure 6:**
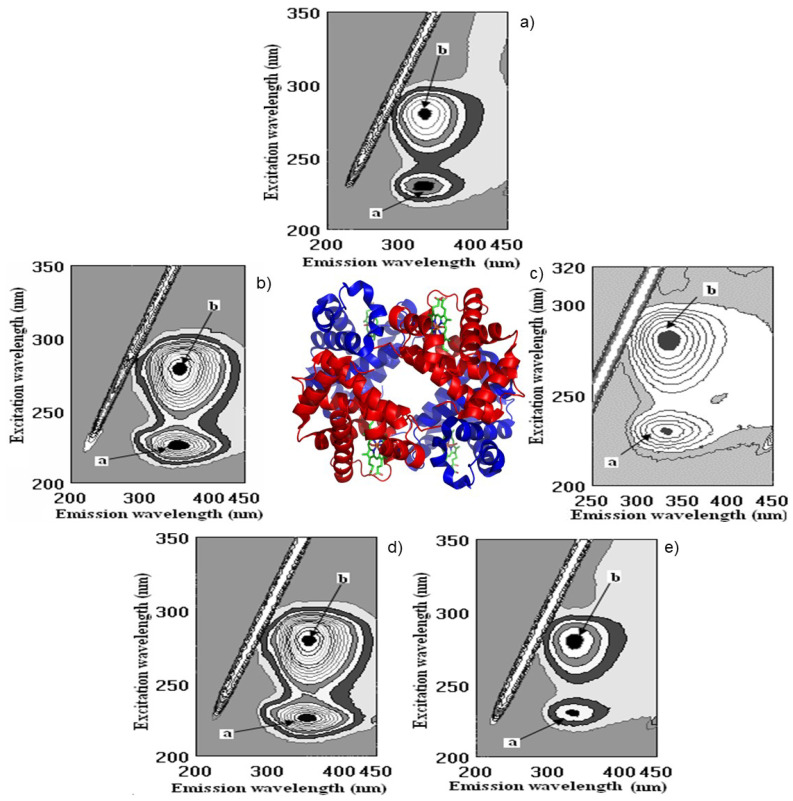
Cross-images of the results obtained from fluorometric measurements in a three-dimensional fluorescence analysis (3D-FL) of the investigated samples after interaction with globulin (Glo) are shown in the following order: (**a**) Glo in the buffer; **(b**) Glo + persimmon wine; (**c**) Glo + pomegranate wine; (**d**) Glo + kiwifruit wine; (**e**) Glo + gallic acid. **a**, **b**, peaks after interaction of investigated samples with Glo.

Determination of polyphenol binding to the main protein in blood human serum is important in human metabolism. The binding of wine polyphenols to human serum proteins was investigated by fluorescence spectroscopy using the quenching of albumin, globulin and fibrinogen fluorescence, and the enhancement of polyphenol fluorescence. As shown above, there are a number of such publications in the field of protein polyphenol interactions, including our recent studies [61,62]. The results of the interaction of wine bioactive compounds with the main human serum proteins are presented in Figure 3, Figure 4, Figure 5 and Figure 6. The cross-images of the results obtained from fluorometric measurements in a three-dimensional fluorescence analysis (3D–FL) of the investigated samples after interaction with fibrinogen show the change in the fluorescence intensity (FI) of fibrinogen (Fgn, Figure 3a) when interaction with wine samples appear (Figure 3b–e) and the shift in wavelength.

The highest decrease in the FI was pomegranate > gallic acid > persimmon> kiwifruit > tannic acid > catechin > ethanol, based on the changes in the intensity of peaks **a** and **b** (Figure 5). The total binding properties of fibrinogen (%) with the wine samples will show the following order: pomegranate (80.5 ± 4.3), gallic acid (65.7 ± 5.4), persimmon (62.2 ± 6.7), kiwifruit (43.6 ± 3.2), catechin (17.6 ± 1.4) and ethanol (2.9 ± 0.3), respectively (Figure 3b–e and Figure 5). Two-dimensional fluorescence measurements (Figure 3f) show the changes in FI from the top of lines with the lowest intensity of pomegranate wine. The calculated binding properties by the results obtained in 2D-FL slightly differed from the results obtained in 3D-fluorescence measurements. Such difference can be explained by the overlap of the obtained intensities of peaks **a** and **b**. In 2D-FL, is peak **b** well shown and not peak **a**. Therefore, the binding properties (%) were the following for kiwi wine of 22.0; persimmon wine of 35.5; for gallic acid of 40.7; for pomegranate wine of 59.1 (Figure 3f, lines from the top 2, 3, 4, 5, respectively).

The changes in the intensities of albumin (Alb, Figure 4a) after interaction with pomegranate, persimmon, gallic and kiwifruit wines are shown in Figure 4b–e and Figure 5.

The highest decrease in FI is shown in the following line: pomegranate > kiwifruit > persimmon > gallic acid > catechin > ethanol (Figure 4 and Figure 5). The total binding properties of Alb (%) with investigated samples the following calculations were carried out as: pomegranate (51.1 ± 3.9), kiwifruit (37.7 ± 3.8), persimmon (30.7 ± 2.9), gallic acid (23.6 ± 2.3), catechin (13.2 ± 1.1), ethanol (2.6 ± 0.3), respectively (Figure 4b–e and Figure 5).

The highest decrease in FI after the interaction of globulin (Glo, Figure 5a), based on the intensity of peaks **a** and **b**, showed the following results: pomegranate > catechin > gallic > kiwi > persimmon > ethanol (Figure 5 and Figure 6).

The highest decrease in FI after the interaction of globulin (Glo, Figure 6a), based on the intensity of peaks **a** and **b** showed the following results: pomegranate > catechin > gallic > kiwi > persimmon > ethanol (Figure 5 and Figure 6).

The binding of Glo (%) with the bioactive substances of wine samples was the following: pomegranate (68.8 ± 5.8), gallic acid (45.5 ± 3.9), kiwifruit (36.5 ± 4.6), catechin (34.8 ± 2.9), persimmon (22.7 ± 2.3), ethanol (3.1 ± 0.3), respectively, Figure 5 and Figure 6b,c,e.

The results of binding properties of main human proteins with fruit wines showed a correlation between the antioxidant activities and their quenching. We hypothesized that if polyphenols of wine samples highly bind the proteins, then it is possible that there will be an interaction of these substances with drugs. Epidemiological and clinical studies highlighted that regular and moderate wine consumption (one to two glasses a day) is associated with a decreased incidence of cardiovascular disease [37]. However, there are discrepancies regarding the specific effects of different types of beverages (wine, beer and spirits) on the cardiovascular system and cancer, and also whether the possible protective effects of alcoholic beverages are due to their alcoholic content (ethanol) or their non-alcoholic components (mainly polyphenols). The presented results showed that the binding properties of ethanol were about 2–3% in comparison to the high binding properties of wine samples (22–80%). The present results are in line with others [4]. Wine drinking has to be in a moderate way in spite of the matter that not alcohol but polyphenols are the main substances participating in their binding. The bioaccessibility of most polyphenols decreased as the drinking amount increased, indicating that drinking larger volumes of wine did not increase the bioaccessibility of polyphenols. Hence, in order to let wine polyphenols play their function for human health, there is still a need for a moderate consumption amount of wine, and drinking after a meal is better [63].

**Figure 7:**
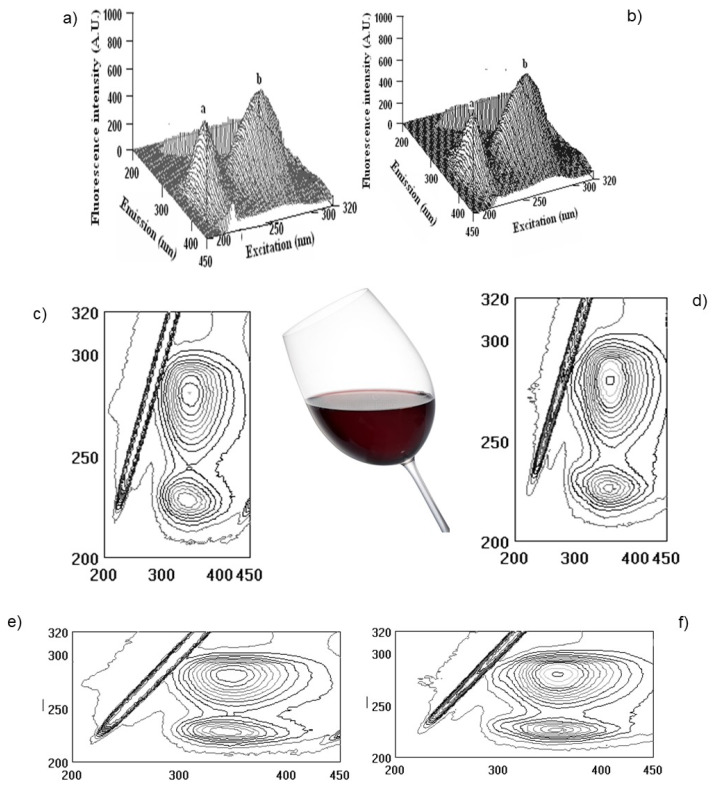
Three-dimensional fluorescence (3D–FL) spectra of the interaction of albumin (Alb) with pomegranate wine extracts (**a**,**b**), 3D-FL of Alb with pomegranate vintage 2006, 2020, respectively; (**c**,**d**) cross images of the results obtained from 3D–FL of the interaction of Alb with pomegranate vintage 2006, 2020, respectively; (**e**,**f**) 3D-FL counter maps of the interaction of Alb with pomegranate vintage 2006, 2020 (for interpretation of the references to color in this figure legend, the reader is referred to the web version of this article). **a**, **b**, peaks after interaction of investigated samples with Alb.

Following this line and the obtained results, it was shown that red fruit pomegranate wine has higher bioactivity in comparison with white fruit wines kiwifruit and persimmon. Two additional samples of pomegranate wine of vintage 2006 (Pomeg2006) and vintage 2020 (Pomeg2020) were compared not only using FTIR but also fluorescence spectra (Figure 7).

The measurements were carried out at the initial albumin (Alb) with λ_ex_/λ_em_ (nm/nm) = 228/353 and 280/357 with fluorescence intensity (FI, arbitral units) = 643.0 and 920.1 for peaks **a** and **b**, respectively. After interaction with Pomeg2006, a change was found in λ_ex_/λ_em_ (nm/nm) = 228/341 with FI = 486.5 for peak **a**, and λ_ex_/λ_em_ (nm/nm) = 281/350 with FI = 659.5 for peak **b**. The calculated binding property (BP, %) = 24.3 ± 1.7 for peak **a** and 28.3 ± 1.9 for peak **b**. The total binding property was 52.6 ± 3.2% (Figure 7a,c,e). After interaction with Pomeg2020, peak **a** showed the following values: λ_ex_/λ_em_ (nm/nm) = 226/337 with FI = 517.2 and peak **b** of λ_ex_/λ_em_ (nm/nm) = 281/358 with FI = 742.2. The obtained binding property (BP, %) of peak **a** was 19.6 ± 1.1 and of peak **b** of 19.3 ± 1.1, and the total quenching of Pomeg2020 was about 38.9 ± 2.8 (Figure 7b,d,f). For the sample of vintage 2022 (Pomeg2022, Table 1, Figure 4 and Figure 6), the total binding of 51.1 ± 3.9% was similar to the calculated for Pomeg2006. The value of polyphenols (mg GAE/L) for Pomeg2022 (Table 1) was 1707.3 ± 11.4 mg GAE/L with a binding of 51.1 ± 3.9%. This estimation was similar to Pomeg2006 (1748.6 ± 12.2 mg GAE/L and 52.6 ± 3.2%). Oppositely, the sample of Pomeg2020 showed lower data (with polyphenols of 1249.9 ± 9.8 mg GAE/L and 38.9 ± 2.8%) than Pomeg2006 and Pomeg2022. As can be seen from the presented data, the binding properties were directly coordinated with the amount of polyphenols. The same relationship between the polyphenols, antioxidant and binding properties was obtained for the interaction with globulin and fibrinogen of different vintages of pomegranate wines (data omitted). The presented data after FTIR, NMR and fluorescence measurements can be used as well as a fingerprint for different vintages.

### 2.5. Molecular Docking Study

**Figure 8:**
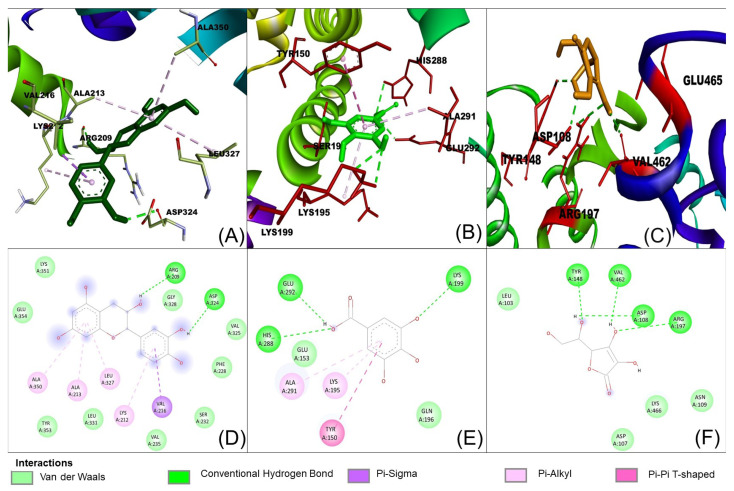
Interaction of ligands with human serum albumin. (**A**–**C**) represent 3D representation of the catechin, gallic acid and vitamin C with HSA. The 2D representation of the ligands showing different types of interaction with the HSA (**D**): catechin; (**E**): gallic acid and (**F**): vitamin C. The interaction types are represented with different color codes.

The possible binding information of catechin, gallic acid and vitamin C to HSA was predicted by molecular docking. Figure 8 shows the interaction of ligands to the HSA in their binding pocket. The binding energies of catechin, gallic acid and vitamin C to HSA were −7.2, −7.8 and −5.8 kJ/mol, respectively.

The result confirmed that the three ligands were bound around the binding site at different positions. Catechin formed two hydrogen bonds with ARG209 and ASP324. The hydrogen bond distances were 2.29 and 2.21 Å, respectively. In contrast, gallic acid formed three hydrogen bonds with Lys 199, His 288 and Glu 292. The hydrogen bond distances were 5.55, 2.95 and 2.0 Å, respectively. Vitamin C showed four hydrogens with Asp 108, Tyr 148, Arg 197 and Val 462 and hydrogen bond distances were 2.30, 2.00, 2.95. 3.1 Å, respectively. The ligands also showed other interactions like pi–alkyl, pi–pi and van der Waals (Table 4).

However, catechin and gallic acid showed a pi–alky interaction with Lys 212 and 195, respectively. Additionally, catechin established a pi–sigma interaction with Val 216. Gallic acid showed a pi–pi T-shaped interaction with Tyr150. From the binding process, it was observed that gallic acid had a stronger binding affinity compared to catechin and vitamin C. The stronger binding affinity of gallic acid may be due to three hydrogen bonds and pi–pi interactions [64].

In conclusion, three types of fruit wines were investigated and compared using different advanced analytical methods. A comparison of the results of the traditional spectral investigation showed that pomegranate wines possessed a higher amount of bioactive substances and antioxidant activities, followed by kiwifruit and persimmon wines. The high antioxidant activities of fruit wines make them an additional source of functional foods. This study indicates that pomegranate red wine has a composition of a higher amount of anthocyanins and phenolic acids and can interact with the key regions of human serum proteins to enhance and increase biological and binding activities. The studied wines interacted with human serum proteins with different binding affinities that were directly related to their antioxidant properties. The highest binding abilities were in pomegranate, followed by kiwi and persimmon wines. This report provides useful information for the future designing of healthy food on the basis of polyphenols and their bioactivity. Polyphenols are hydrophilic molecules, playing the role of blood transport proteins in their delivery to tissues. Therefore, high bioactivity and quenching abilities are important for health and nutritional properties. FTIR, NMR and fluorescence spectra of wines showed the variations among wines, which makes these methods additional tools for the authentication of wines.

## 3. Materials and Methods

### 3.1. Materials

The chemicals 6-Hydroxy-2,5,7,8-tetra-methylchro man-2-carboxylic acid (Trolox), catechin, tannic acid, gallic acid, human serum albumin (Alb), fibrinogen (Fng), globulin (Glo), 2, 4, 6-tripyridyl-s-triazine (TPTZ) aluminium chloride, potassium peroxydisulfate and 2, 2′-azino-bis (3-ethyl-benzothiazoline-6-sulfonic acid) diammonium salt radical cation (ABTS), chloride dihydrate, sodium hydroxide, hydrochloric acid (37% *w*/*w*), phosphate buffer and Folin–Ciocalteu reagent (FCR) were purchased from Sigma (St. Louis, MO, USA) and Fluka Chemie GmbH (Buchs, Switzerland). Standard phenolics were dissolved in methanol (1 mg/mL). All reagents and chemicals were of analytical grade.

All NMR chemicals, including 3-trimethylsilylpropanoic acid (TSP), potassium phosphate monobasic (KH_2_PO_4_), methanol-d4 (CD_3_OD, 99.8%), sodium deuterium oxide (NaOD) and deuterium oxide (D_2_O, 99.9%), were purchased from Merck (Darmstadt, Germany).

### 3.2. Wine Samples

Wines were bought in Israel and South Korea. Pomegranate dry wine with 13.8% alcohol of 2022 vintage was purchased from Rimon Wineries, Israel. Pomegranate wines of vintages 2006 and 2020 were purchased as well in Israel. Persimmon wine (Persimun wine (Regular)) with 12.0% alcohol was delivered from Agricultural Corporation Cheongdo Persimun Wine, Cheongdo, Gyeongsang buk-do, Korea. Kiwi (Darae) wine with 8.0% alcohol was made from kiwis (chamdaraein Korean). The sample quantity was the following: each kind of wine was purchased in the amount of five samples in several places, but from the same year of vintage and showed the same shelf life.

### 3.3. Analyses of Bioactivity in Wine Samples

The total phenolic amount (TP) was measured by using the Folin–Ciocalteu method [41]. After using 250 μL of wine mixed with 1000 μL of sodium carbonate (7.5%) and 1250 μL of Folin–Ciocalteu’s (10% in water) reagent, the mixture was incubated for 15 min at 50 °C in the dark (water bath) and measured at 765 nm, using a spectrophotometer (Hewlett-Packard, model 8452A, (Rockville, MD, USA). Gallic acid was used as the standard, and the results were expressed as milligrams of gallic acid equivalent per liter (mg GAE/L). The anthocyanin content (AC) in wines was measured in aliquots of 250 μL of the wine sample, which was poured into a tube with 2 mL of potassium chloride solution (0.025 M) and adjusted to pH 1 with concentrated HCl. The mixture was incubated at room temperature for 20 min. In another tube, 250 μL of wine was mixed with 2 mL of sodium acetate solution (0.4 M, pH 4.5) and incubated at room temperature for 20 min. The absorbance of an aliquot of 300 μL of each wine sample was measured at 520 and 700 nm. The results were expressed as milligrams of cyanidin 3-glucoside equivalent per L (mg C3G/L) [65,66].

The total tannins (TNs) were estimated by using spectrophotometric measurements of 0.5 mL of wine, where 3 mL of a 4% methanol vanillin solution and 1.5 mL of concentrated hydrochloric acid were added. The mixture was allowed to stand for 15 min. The absorption of the samples and a blank against water was measured at 500 nm [38].

Total ascorbic acid content (TAAC, mg ascorbic acid (AA) per L) was evaluated in water wine extracts, where 100 mg of the freeze-dried wine sample was extracted with 5 mL water. Then, the cupric ion reducing antioxidant capacity (CUPRAC) method was conducted and formed bis (Nc)-copper (I) chelate was determined spectrophotometrically at 450 nm [39].

Some bioactive compounds, such as catechin and gallic acid, were determined with a high-performance liquid chromatography (HPLC) system [67]. A volume of 50 mL of each of the wine samples was extracted three times with 25 mL of diethyl ether and then three times with 25 mL of diethylacetate, and the organic fractions were combined. After 30 min of drying with anhydrous Na_2_SO_4_, the extract was filtered through a Whatman-40 filter and evaporated to dryness in a rotary evaporator. The residue was dissolved in 2 mL of methanol/water (1:1, *v*/*v*) and analyzed by using high-performance liquid chromatography (HPLC). A Waters (Milford, MA, USA) chromatograph equipped with a6 00-MS controller, a 717 plus autosampler and a 996 photodiode-array detector was used. A gradient of solvent A (water/acetic acid, 98:2, *v*/*v*) and solvent B (water/acetonitrile/acetic acid, 78:20:2, *v*/*v*/*v*) was applied to a reverse-phase Nova-pack C18 column (30 cm × 3.9 mm internal diameter (I. D.)) as follows: 0–55 min, 80% B linear, 1.1 mL/min; 55–57 min, 90% B linear, 1.2 mL/min; 57–70 min, 90% B isocratic, 1.2 mL/min; 70–80 min, 95% B linear, 1.2 mL/min; 80–90 min, 100% B linear, 1.2 mL/min; 90–120 min. For the HPLC analysis, an aliquot (50 µL) was injected into the column and eluted at the temperature of 20 °C. The quantitative values are given in Table 1.

The 2,2′-azino-bis (3-ethyl-benzothiazoline-6-sulfonic acid) diammonium salt (ABTS) radical cation was formed by the ABTS solution (7 mM) with potassium persulfate (2.45 mM) in distilled water at room temperature at 16 h before use. A working solution (ABTS reagent) was diluted to obtain absorbance values of 0.7 at 734 nm and equilibrated at 30 °C. After the addition of ABTS solution, the absorbance reading was taken 1 min after the initial mixing and for up to 6 min; the percentage inhibition of absorbance was calculated with reference to a Trolox calibration curve and evaluated as mM Trolox equivalent/L of wine [65].

The antioxidant capacity in the wines was measured by using the ferric reducing ability of plasma (FRAP) in 24 µL of the sample, which was mixed with 180 µL of FRAP reagent (TPTZ 10 mM in HCl 40 mM, iron chloride hexahydrate 20 mM, acetate buffer 0.3 M, pH 3 in a ratio of 1:1:10, prepared daily). The reaction was carried out at 37 °C, and the absorbance was measured at 595 nm every min for 30 min [68].

### 3.4. Fourier Transform Infrared Spectra of Polyphenols

Total phenols in the investigated fruit wine extracts were studied by IR spectroscopy. A Nicolet iS 10 Fourier transform infrared (FT-IR) spectrometer (ThermoScientific Instruments LLC, Madison, WI, USA), with the smart iTRTM attenuated total reflectance (ATR) accessory was used to record IR spectra. The spectra were also scanned in the 500–4000 cm^−1^ range with a spectral resolution of 4 cm^−1^ and plotted as % transmittance versus wave numbers. Each evaluated spectrum is a mean of 32 scans [69].

### 3.5. ^1^H NMR Spectroscopy

Each extract of wine samples was dissolved in 700 μL of dimethyl sulfoxide (DMSO). Extraction was also carried out with methanol/chloroform/water at a 2:2:1 volumetric ratio. Samples were kept at 4 °C for 1 h and then centrifuged for 20 min at 11,000× *g* at 4 °C. The upper hydroalcoholic phase was carefully separated and dried under a N_2_ flow. The dried phase was stored at −80 °C until the NMR analysis. Both (375 μL) CD_3_OD solvent and KH_2_PO_4_ buffer in D_2_O (pH 6.0), containing 0.1% of TSP, were added to each sample [70].

### 3.6. Fluorometric Studies

The properties of bioactive substances in wines were determined by using three-dimensional (3D-FL) fluorescence (model FP-6500, Jasco spectrofluorometer, serial N261332, Tokyo, Japan). The 3D-FL was measured at emission wavelengths between 200 and 795 nm, and the initial excitation wavelength was 200 nm. For comparison of the obtained results, catechin and gallic acid were used [71]. Standard phenolic solutions, such as gallic acid and catechin, were prepared daily by dissolving at a concentration of 10 mM in methanol and then diluting with 10 mM phosphate buffer at pH 7.4. The initial fluorescence intensities of fibrinogen, albumin and globulin were measured before their interactions with the investigated wines. The decreases in the fluorescence intensities were used in the estimation of the binding activities.

### 3.7. Molecular Docking Study

The ligands such as catechin, vitamin C and gallic acid found in the extract of pomegranate, persimmon and kiwifruit were downloaded from the PubChem database (https://pubchem.ncbi.nlm.nih.gov/, accessed on 21 May 2023). The X-ray crystal structures are available with a protein databank (PDB) (www.rcsb.org, accessed on 21 May 2023). The target protein for the present study human serum albumin (HSA) was downloaded from PDB (7VR0.pdb) with a resolution of 1.98 Å. Before docking, the ligands and proteins were prepared using the Autodock Tool program. The downloaded protein was energy minimized, the Gasteiger charges were added and proteins were saved in pdbqt format. Ligands obtained from the PubChem database were prepared with the addition of Kolman and Gasteiger charges and ligands were saved in pdbqt. The grid box size of 50.9 Å × 32.3 Å × 25 Å (X, Y, Z) and with exhaustiveness of 8 was applied for the proteins. The ligands prepared were loaded into the workspace of the PyRx virtual screening tool with the Auto Dock VINA Wizard. The two-dimensional (2D) and three-dimensional (3D) graphical depictions of all the complexes were accomplished using Discovery Studio client Visualizer v21.1 [72,73].

### 3.8. Data Analysis

All data obtained were calculated on the basis of a statistical analysis of Duncan’s multiple range test. Values were mean s ± SD per liter of 25 measurements, representing the commercial status of the wines and their replicates. Five replications of five wine samples were used. To determine the statistical significance at the 95% interval of reliability, a one-way analysis of variance (ANOVA) was used, Graph-Pad Prism v.3.02 (GraphPad Software, San Diego, CA, USA; ANOVA, Student’s *t*-test).

## 4. Conclusions

Three types of fruit wines were investigated and compared using different advanced analytical methods. A comparison of the results of the traditional spectral investigation showed that pomegranate wines possessed a higher amount of bioactive substances and antioxidant activities, followed by kiwifruit and persimmon wines. The high antioxidant activities of fruit wines make them an additional source of functional foods. This study indicated that pomegranate red wine has a composition of a higher amount of anthocyanins and phenolic acids and can interact with the key regions of human serum proteins to enhance and increase biological and binding activities. The studied wines interacted with human serum proteins with different binding affinities, which were directly related to their antioxidant properties. The highest binding abilities were in pomegranate, followed by kiwi and persimmon wines. This report provides useful information for the future designing of healthy food on the basis of polyphenols and their bioactivity. Polyphenols are hydrophobic molecules, playing the role of blood transport proteins in their delivery to tissues. Therefore, high bioactivity and quenching abilities are important for health and nutritional properties. FTIR, NMR and fluorescence spectra of wines showed the variations among wines, which makes these methods an additional tool for the identification of wines.

## Figures and Tables

**Table 1:** Antioxidant and binding properties of fruit wines/L with human serum proteins: albumin (Alb), globulin (Glo) and fibrinogen (Fgn).

Indices	Pomegranate	Persimmon	Kiwifruit	Ethanol
Polyphenols, mg GAE	1707.3 ± 11.4 ^a^	917.9 ± 8.4 ^b^	1325.8 ± 13.9 ^ab^	
Anthocyanins, mgCGE	88.2 ± 4.1 ^a^	79.2 ± 3.6 ^ab^	66.7 ± 2.2 ^b^	
Tannins, mg	182.7 ± 3.3 ^b^	337.8 ± 8.2 ^a^	157.1.6 ± 4.1 ^c^	
Catechin, mg	17.4 ± 1.3 ^a^	5.9 ± 0.4 ^b^	7.9 ± 0.8 ^ab^	
Vitamin C, mg AA	16.1 ± 1.4 ^b^	9.9 ± 0.8 ^b^	64.6 ± 5.3 ^a^	
ABTS, mM TE/L	20.2 ± 1.9 ^a^	11.4 ± 0.9 ^c^	16.5 ± 1.7 ^b^	
Total binding to Alb, %	51.1 ± 3.9 ^a^	30.7 ± 2.9 ^b^	37.7 ± 3.8 ^ab^	2.6 ± 0.3 ^c^
Total binding to Glo, %	68.8 ± 5.8 ^a^	22.7 ± 2.3 ^c^	36.5 ± 4.6 ^b^	3.1 ± 0.3 ^c^
Total binding to Fgn, %	80.5 ± 4.3 ^a^	62.2 ± 6.7 ^ab^	43.6 ± 3.2 ^b^	2.9 ± 0.3 ^c^
FRAP, mMTE/L	7.4 ± 0.9 ^a^	3.4 ± 0.1 ^ab^	5.9 ± 0.8 ^b^	
Gallic acid, mg	108.8 ± 5.9 ^a^	48.1 ± 3.1 ^ab^	64.7 ± 4.2 ^b^	

Values are means ± SD of 5 measurements; means within a raw with the different superscripts or without superscripts are statistically different (*p* < 0.05; Student’s *t*-test). Abbreviations: GAE, gallic acid equivalent; ABTS, 2, 2′-azino-bis (3-ethyl-benzothiazoline-6-sulfonic acid) diammonium salt radical cation; TE, Trolox equivalent; FI, fluorescence intensity; A. U., arbitral units; per g dry weight (DW); FRAP, ferric reducing ability of plasma. FI of HSA in water according to peak **a** is equal to 570.21 ± 9.2; peak **b** is equal to 852.40 ± 11.3. ^c^ is used for statistical evaluation in Table 1.

**Table 2:** Correlation of FTIR shifts and comparison of wine samples.

Samples	Correlation	Q-Check Regions, cm^−1^
Pomegr–Kiwi	0.5559	3627.5–3285.5
	0.5426	1697.5–1591.0
	0.5940	1204.5–1113.3
	0.8055	1094.5–1053.5
	0.5629	981–905.5
Pomegr–Persimm.	0.5795	3085.5–2906.0
	0.6523	1739.5–1568.5
	0.8645	1314.0–1221.5
	0.8762	1125.5–1037.5
	0.8068	1011.5–906.5
Kiwi–Persimm.	0.5170	3568.5–3138.0
	0.6663	1823.0–1665.0
	0.4315	1283.5–1139.0

**Table 4:** The binding affinity and interaction of the ligands with the amino acid of human serum albumin.

Ligand	Binding Affinity (Kcal/mol)	Amino Acids Involved in the Interaction (H-Bond)	Other Interactions
Catechin	−7.2	ARG209 and ASP324	Lys 212 (Pi-alkyl), Val 216 (Pi–sigma), Phe 228, Ser 232, Val 235, Val 325, Leu 331, Lys 351, Tyr 353, Glu 354 (van der Waals)
Gallic acid	−7.8	Lys 199, His 288 and Glu 292	Lys 195 and Ala 291 (Pi-alkyl), Tyr 50 (Pi-Pi T-shaped)
Vit C	−5.8	Asp 108, Tyr 148, Arg 197 and Val 462	Leu 103, Asn 109, Asp 107, Lys 466

## Data Availability

The data presented in this study are available upon request from the corresponding author. The data are not publicly available for privacy reasons.

## Sample Availability

Samples of the compounds are not available from the authors.
